# Perioperative Anesthetic Management of a Child With Dystrophic Epidermolysis Bullosa Undergoing Hand Contracture Release

**DOI:** 10.7759/cureus.110096

**Published:** 2026-06-02

**Authors:** Norin Qashifa, Mekha Elizabeth Mathew, Venkatesh Selvaraj, Jayaraman V

**Affiliations:** 1 Anesthesiology, Sri Ramachandra Institute of Higher Education and Research, Chennai, IND

**Keywords:** contracture release surgery, difficult airway, dystrophic epidermolysis bullosa, microstomia, pediatric anesthesia, perioperative management, skin fragility, video laryngoscopy

## Abstract

Dystrophic epidermolysis bullosa (DEB) is a rare inherited disorder characterized by extreme skin and mucosal fragility, posing significant anesthetic challenges. We report the perioperative management of an 11-year-old boy with severe DEB and microstomia undergoing hand contracture release under general anesthesia. He presented with mitten-hand deformities, restricted mouth opening (1.2 cm), limited neck extension, anemia, and difficult intravenous access. Preoperative optimization included a blood transfusion. Meticulous precautions were taken to prevent skin trauma, including padded positioning, modification of monitoring techniques, and hydrocolloid dressings beneath adhesives. Inhalational induction with sevoflurane was performed while maintaining spontaneous ventilation. Fiberoptic intubation was unsuccessful due to restricted oropharyngeal space; tracheal intubation was achieved using a C-MAC video laryngoscope with continuous para-oxygenation through a size 5 nasal airway gently inserted into the child's left nostril. A tourniquet was avoided. Anesthesia and recovery were uneventful. This case highlights the importance of careful planning, gentle tissue handling, airway preparedness, and individualized anesthetic strategies to minimize perioperative complications in children with DEB.

## Introduction

Epidermolysis bullosa encompasses a group of inherited mechanobullous disorders in which minor trauma results in blistering of the skin and mucous membranes [1]. Dystrophic epidermolysis bullosa (DEB) is caused by a mutation in the gene encoding type VII collagen, an essential component of anchoring fibrils at the dermal-epidermal junction [2]. The clinical presentation ranges from mild blistering to severe, generalized forms with significant extracutaneous manifestations [3]. Patients often develop progressive deformities such as mitten-hand and foot contractures, microstomia from oral scarring, esophageal strictures, and dental anomalies [1,3].

The anesthetic management of children with DEB is complex. The extreme fragility of the skin and mucosa necessitates meticulous care to prevent pressure-induced or friction-related blistering [4]. Standard procedures such as applying monitoring equipment, securing intravenous access, and positioning the patient require significant modifications [4]. Airway management is particularly challenging due to microstomia, limited neck mobility from scarring, and the risk of bleeding in the oropharynx and trachea during instrumentation [5].

## Case presentation

**Figure 1:**
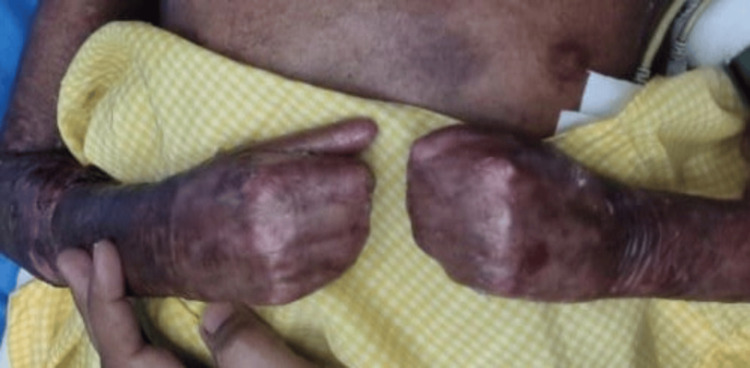
Severe dystrophic epidermolysis bullosa with loss of nails and fusion of the fingers resulting in mitten-hand deformity

An 11-year-old boy presented with bilateral mitten-hand deformities. He had a history of recurrent fluid-filled blisters and erosions involving the skin since birth, progressively affecting multiple body regions. From the age of three years, he developed loss of nails with progressive fusion of the fingers and toes (Figure 1). He was subsequently diagnosed with DEB, and antigen mapping demonstrated a complete absence of type VII collagen. He had not received any definitive treatment until the present admission, when he was scheduled for bilateral hand contracture release.

**Figure 2:**
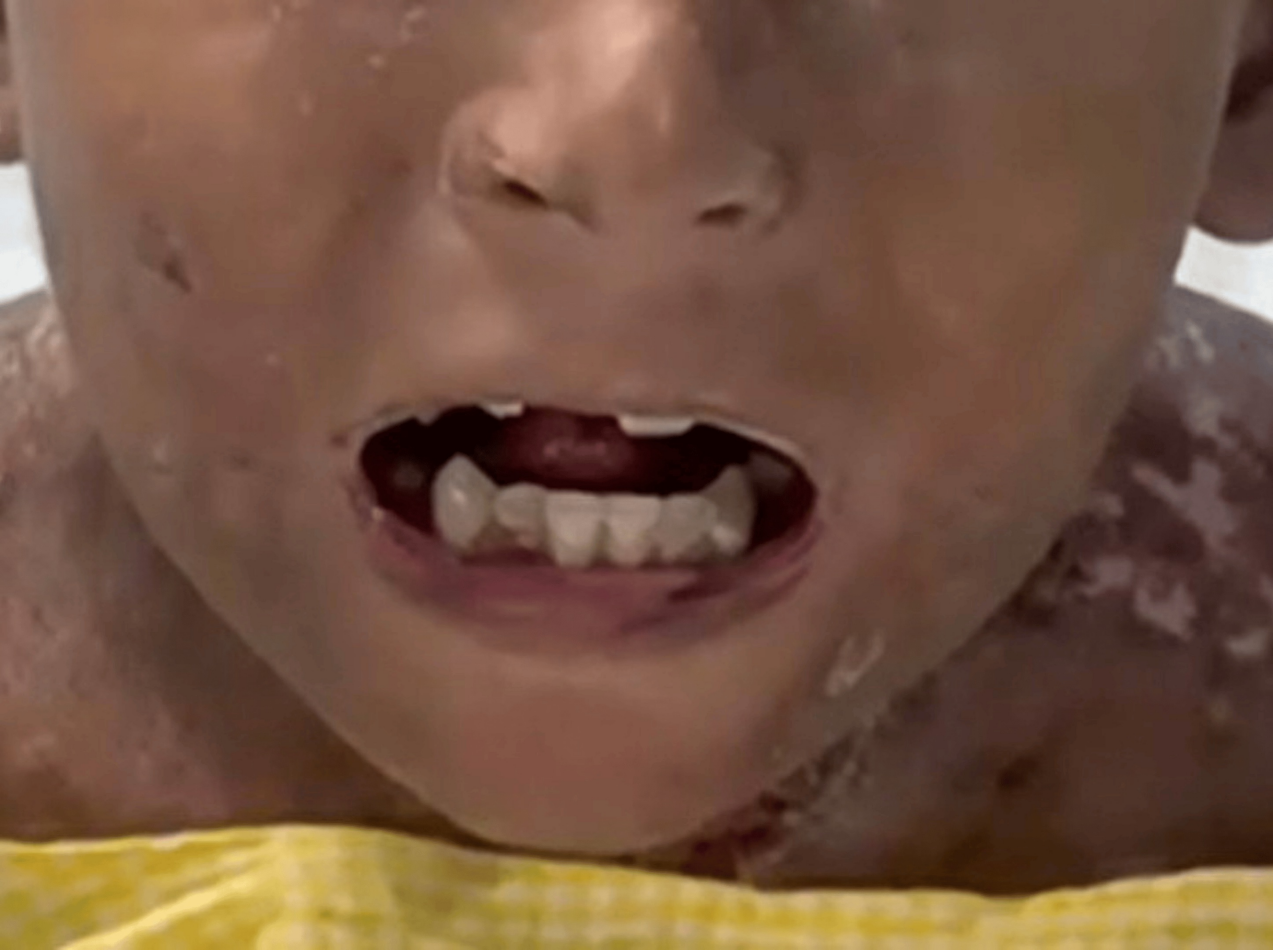
Restricted mouth opening resulting from chronic scarring and contracture of the oral tissues

The child had developed microstomia secondary to recurrent oral blistering. Mouth opening was severely restricted, measuring approximately 1.2 cm (Figure 2). He also had multiple broken teeth. Neck extension was limited due to contractures resulting from scarring.

The child weighed 18 kg and was 120 cm tall. On admission, the hemoglobin level was 6.5 g/dL. The anemia was nutritional and due to iron deficiency. Serum iron and ferritin levels were reduced, and the peripheral smear showed microcytic hypochromic anemia. Moreover, multiple sites of skin blistering and bleeding may also have contributed to anemia due to the constant loss of small amounts of blood. Two units of packed red blood cells (300 mL each) were transfused. Post-transfusion hemoglobin increased to 11.8 g/dL.

**Figure 3:**
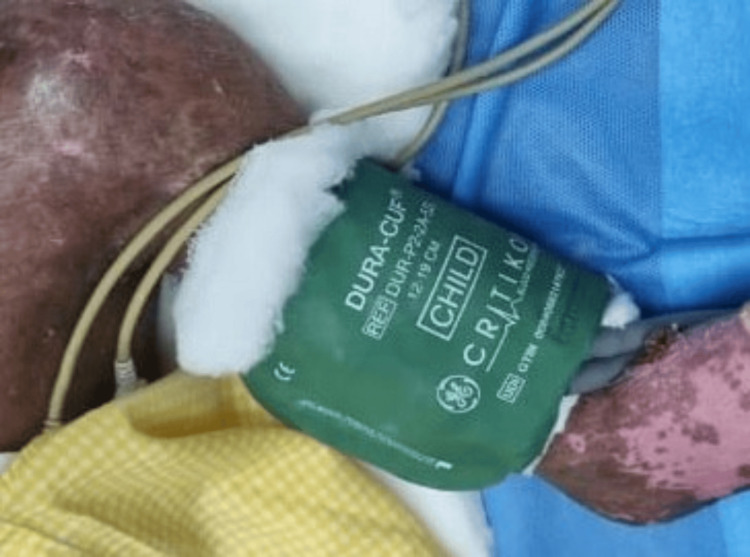
Adequate padding was provided at all pressure points and between the blood pressure cuff and the skin to prevent skin trauma

After obtaining informed parental consent, the child was taken to the operating theater. The operating table was fully padded with cotton to minimize skin trauma, and the upper arm was padded before applying the noninvasive blood pressure cuff (Figure 3).

**Figure 4:**
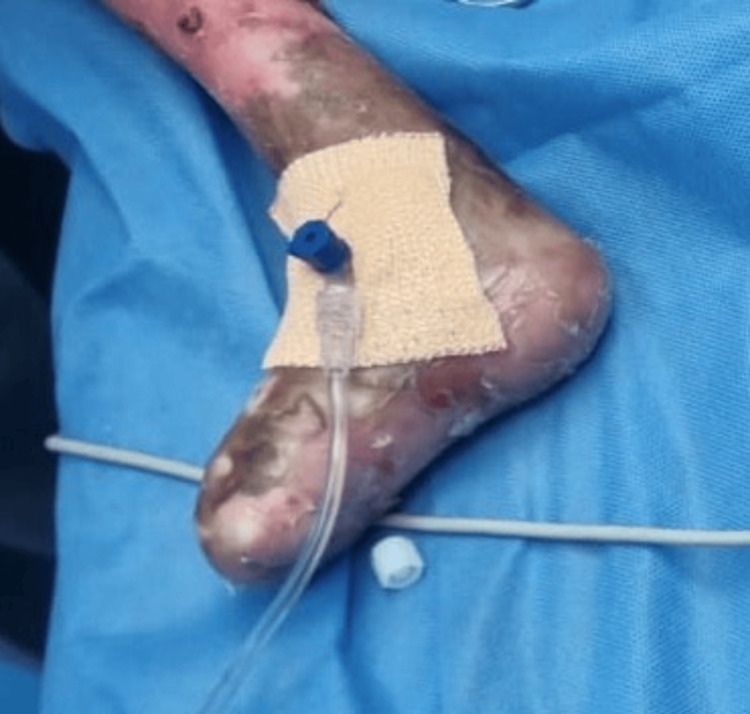
Difficult intravenous cannulation. The IV line was fixed in place with a flexible adhesive bandage, with a hydrocolloid dressing placed between the skin and the adhesive to prevent skin tearing

The pulse oximeter probe was placed on the earlobe. Inhalational induction was performed with sevoflurane while maintaining spontaneous ventilation. Adequate padding was provided around the face mask to decrease mucosal injury. Once an adequate depth of anesthesia was achieved, intravenous access was secured in the right saphenous vein using a 22-gauge cannula under ultrasound guidance and fixed with a flexible adhesive bandage after placing a layer of hydrocolloid (Duoderm) dressing between the skin and the adhesive to prevent skin injury (Figure 4).

**Figure 5:**
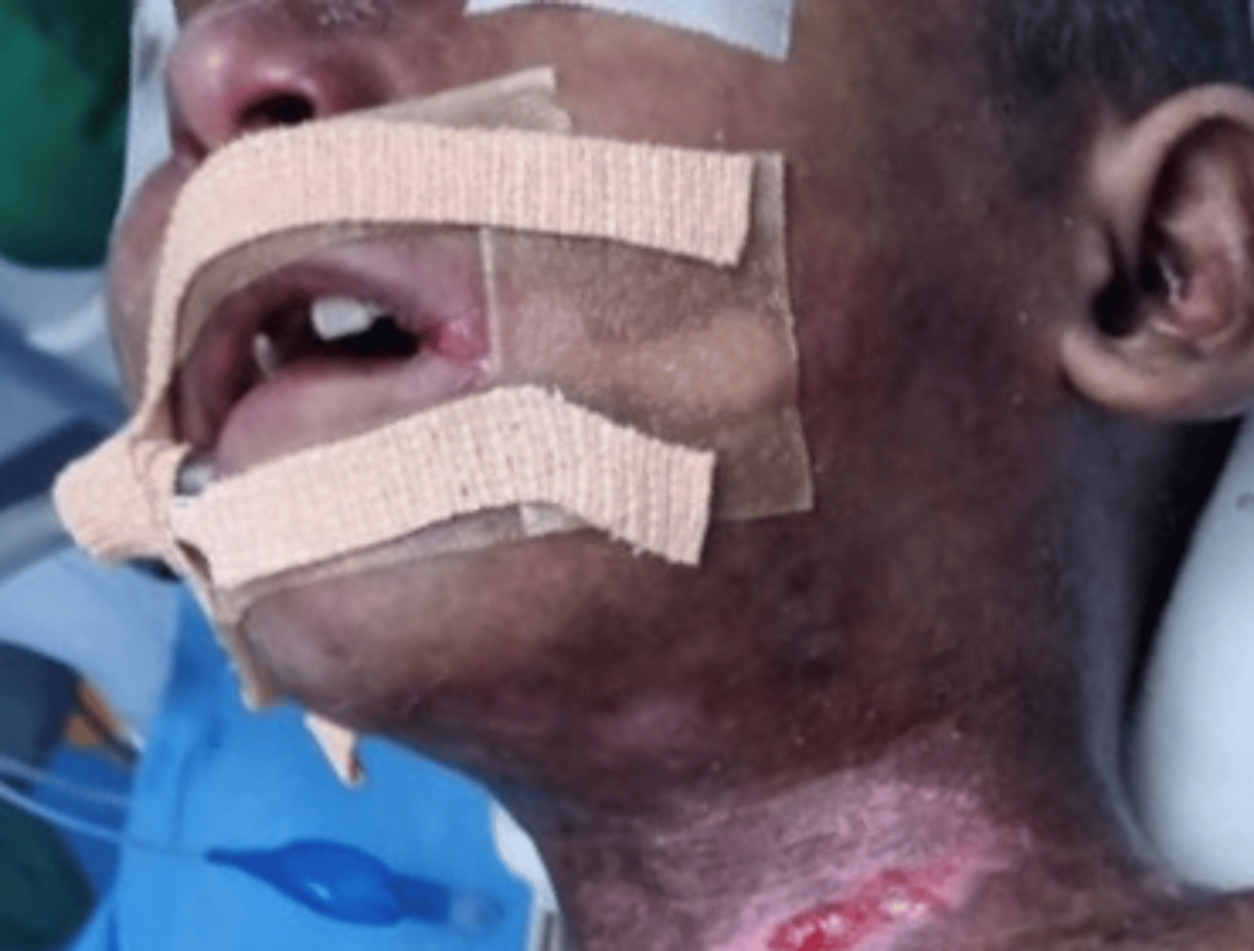
Difficult airway. The flexometallic endotracheal tube was secured around the child's mouth using adhesive bandages, with a hydrocolloid dressing placed between the skin and the adhesive for skin protection

Intravenous fentanyl (1 µg/kg) and propofol (1 mg/kg) were administered prior to the attempt at intubation. A lubricated size 5 nasopharyngeal airway was inserted through the left nostril to facilitate continuous para-oxygenation with 100% oxygen. The child was still spontaneously breathing as fiberoptic intubation was initially attempted; however, it was unsuccessful due to restricted oropharyngeal space, which prevented advancement of the endotracheal tube to the level of the vocal cords. There was also mucosal bleeding, which obscured visualization. Subsequently, tracheal intubation was accomplished using a C-MAC video laryngoscope with a size 2 blade while maintaining spontaneous ventilation under sevoflurane anesthesia. The glottic view was Cormack-Lehane grade IIa. A size 5 cuffed endotracheal tube was successfully secured (Figure 5). Continuous para-oxygenation via the nasopharyngeal airway with 100% oxygen was maintained throughout airway management.

Neuromuscular blockade was then achieved with atracurium (0.5 mg/kg) and maintained with incremental doses of 0.1 mg/kg. Anesthesia was maintained with sevoflurane in a mixture of oxygen and air. Sevoflurane was maintained at 2% with a total gas flow of 2 L/min of oxygen and air (FiO₂ 50%). Analgesic requirements were managed using titrated doses of fentanyl and intravenous paracetamol. Intraoperatively, vital signs remained stable. A tourniquet was not used due to the fragility of the skin. Blood loss was 150 mL. One unit of packed red blood cells (100 mL) was transfused. The surgery lasted 70 minutes. Left-hand contracture release was planned for a later date. After adequate reversal of neuromuscular blockade with Inj. neostigmine (0.05 mg/kg) and Inj. glycopyrrolate (0.01 mg/kg) and full awakening, the child was extubated. Postoperatively, 5 mg of Inj. morphine was administered in two divided doses, four hours apart. A postoperative block was considered but was not performed due to the extreme fragility of the skin.

## Discussion

Anesthetic management in DEB is complex and requires meticulous planning and gentle handling to prevent iatrogenic injury [4]. Airway management is often the most critical challenge due to microstomia, limited neck mobility, mucosal fragility, and dental abnormalities [3]. Fiberoptic intubation is generally considered the technique of choice [5]; however, bleeding and poor visualization may necessitate adjuncts such as video laryngoscopy, as demonstrated in this case.

Skin protection is paramount throughout the perioperative period. Standard monitoring techniques must be modified with adequate padding, avoidance of adhesives, and minimal pressure [6]. Securing intravenous access can be particularly difficult, and the use of protective dressings beneath fixation materials is essential [6]. Tourniquets should be avoided whenever possible because of the risk of skin necrosis and blister formation [6]. Multimodal analgesia with minimally invasive interventions is preferred in the postoperative period [7]. Early extubation and careful monitoring help reduce the risk of airway trauma and respiratory complications [8,9]. Emergency preparedness and multidisciplinary coordination are essential in managing complications in these patients [10].

## Conclusions

DEB poses unique and significant challenges for the anesthesiologist, especially in pediatric patients requiring surgery. All basic anesthetic interventions, such as IV cannulation, attachment of standard American Society of Anesthesiologists (ASA) monitors, and airway management, become challenging in these children. Thorough preoperative assessment, anticipation of a difficult airway, modification of routine anesthetic techniques, and meticulous skin care are essential. With careful planning and gentle handling, anesthesia can be safely administered while minimizing perioperative morbidity.
